# Bis{2-[3-(dimethyl­amino)propyl­imino­meth­yl]-6-methoxy­phenolato-κ^3^ *N*,*N*′,*O* ^1^}nickel(II)

**DOI:** 10.1107/S1600536808020497

**Published:** 2008-07-09

**Authors:** Hang-Ming Guo, Hong Lin

**Affiliations:** a Jinhua Professional Technical College, Jinhua, Zhejiang Province 321017, People’s Republic of China

## Abstract

The centrosymmetric title complex, [Ni(C_13_H_19_N_2_O_2_)_2_], is a mononuclear nickel(II) complex. The Ni^II^ atom is coordinated by four N atoms and two O atoms of two deprotonated Schiff base ligands, forming a slightly distorted octa­hedral coordination configuration, in which the tertiary N atoms occupy the axial positions.

## Related literature

For related literature, see: Choudhury *et al.* (2001 ▶); Das *et al.* (1997 ▶); Davies *et al.* (1973 ▶); Feng (2003 ▶); Li & Wang (2007 ▶); Pariya *et al.* (1995 ▶).
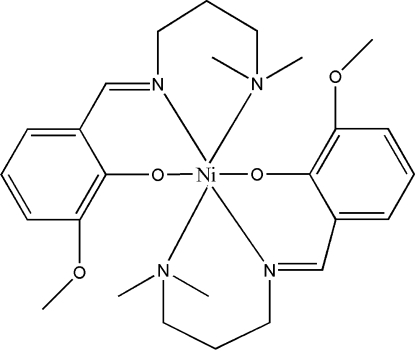


## Experimental

### 

#### Crystal data

[Ni(C_13_H_19_N_2_O_2_)_2_]
*M* *_r_* = 529.31
Triclinic, 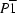

*a* = 7.4758 (15) Å
*b* = 8.5571 (17) Å
*c* = 10.995 (2) Å
α = 78.36 (3)°
β = 73.98 (3)°
γ = 73.73 (3)°
*V* = 643.0 (2) Å^3^
*Z* = 1
Mo *K*α radiation
μ = 0.79 mm^−1^
*T* = 296 (2) K
0.35 × 0.28 × 0.26 mm

#### Data collection

Bruker APEXII area-detector diffractometer
Absorption correction: multi-scan (*SADABS*; Sheldrick, 1996 ▶) *T*_min_ = 0.766, *T*_max_ = 0.814
10449 measured reflections
2937 independent reflections
2727 reflections with *I* > 2σ(*I*)
*R*_int_ = 0.020

#### Refinement

*R*[*F* ^2^ > 2σ(*F* ^2^)] = 0.028
*wR*(*F* ^2^) = 0.077
*S* = 1.00
2937 reflections
160 parameters
H-atom parameters constrained
Δρ_max_ = 0.32 e Å^−3^
Δρ_min_ = −0.17 e Å^−3^

### 

Data collection: *APEX2* (Bruker, 2004 ▶); cell refinement: *SAINT* (Bruker, 2004 ▶); data reduction: *SAINT*; program(s) used to solve structure: *SHELXS97* (Sheldrick, 2008 ▶); program(s) used to refine structure: *SHELXL97* (Sheldrick, 2008 ▶); molecular graphics: *SHELXTL* (Sheldrick, 2008 ▶); software used to prepare material for publication: *SHELXL97*.

## Supplementary Material

Crystal structure: contains datablocks I, global. DOI: 10.1107/S1600536808020497/at2571sup1.cif

Structure factors: contains datablocks I. DOI: 10.1107/S1600536808020497/at2571Isup2.hkl

Additional supplementary materials:  crystallographic information; 3D view; checkCIF report

## Figures and Tables

**Fig. 1.**
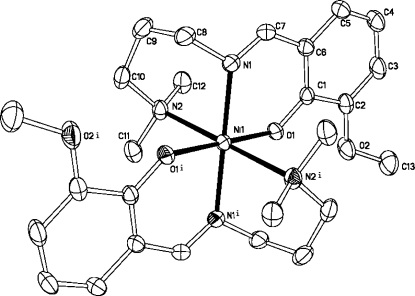
A view of the molecule of (I), showing the atom-labelling scheme, displacement ellipsoids are shown at the 30% probability level. [Symmetry codes: (i) -x + 1,-y,-z + 1]

## supplementary crystallographic information

### Comment

There is considerable interest in the synthesis of multidentate Schiff base ligands for their versatile coordination behavior to metal ions and wide application in biological systems (Das *et al.*, 1997). Metal complexes with tetradentate N_2_O_2_ and tridentate N_2_O Schiff base ligands derived from salicylaldehyde have been well studied in the past, such as [Ni(C_12_H_18_N_2_O_2_)_2_Cl_2_] (Feng, 2003), [Mn(C_18_H_17_N_2_O_4_)] (Davies *et al.*, 1973) and [Ni(Me_2_NCH_2_CH_2_CH_2_N=CHC_6_H_4_O)_2_] (Choudhury *et al.*, 2001). The title complex, [Ni(C_13_H_19_N_2_O_2_)_2_], has a crystallograpic center with the Ni atom situated at the center of (1/2, 0, 1/2). As illustrated in Fig. 1, the center Ni^II^ ion is octahedrally coordinated by two tridentate chelate ligands in a meridional arrangement resulting in a slightly distorted octahedral geometry. The equatorial plane is formed by two imine nitrogen atoms (N1 and N1^i^) and two deprotonated phenolate oxygen atoms (O1 and O1^i^) with the deviation of the metal ion of 0.003 (1) Å. The axial positions are occupied by the tertiary nitrogen atoms (N2 and N2^i^). Like other reported structures, (Li & Wang, 2007; Pariya *et al.*, 1995), the axial Ni(1)—N(2) distance (2.308 (1) Å) is larger than the equatorial Ni(1)—N(1) distance (2.055 (1) Å). The bond angles around the Ni^II^ ion also deviate slightly from the ideal octahedron geometry. Angles involving the atoms in the *trans* positions are 180° but those invoving the *cis*-atoms vary from 81.07 (6)–98.96 (6)°.

### Experimental

3-methoxysalicylaldehyde (2.0 mmol) and 3-dimenthylaminopropylamine (2.0 mmol) in 15 ml of methyl alcohol were stirred for 4 h. NiCl_2_^.^4H_2_O (1.0 mmol) was added and stirred for 10 h. The resulting solution was placed in a refrigerator at 263 K for 10 days, and the crystals were filtered off, giving orange crystals of the title complex for X-ray analysis.

### Refinement

All H atoms were positioned geometrically and constrained to ride on their parent atoms, with C—H distances in the range 0.93 - 0.97 Å, and with *U*_iso_(H) = 1.2 or 1.5*U*_eq_(C).

### Crystal data

**Table:**

[Ni(C_13_H_19_N_2_O_2_)_2_]	*Z* = 1
*M**_r_* = 529.31	*F*_000_ = 282
Triclinic, *P*1	*D*_x_ = 1.367 Mg m^−^^3^
Hall symbol: -P 1	Mo *K*α radiation λ = 0.71073 Å
*a* = 7.4758 (15) Å	Cell parameters from 10453 reflections
*b* = 8.5571 (17) Å	θ = 1.9–27.5º
*c* = 10.995 (2) Å	µ = 0.79 mm^−^^1^
α = 78.36 (3)º	*T* = 296 (2) K
β = 73.98 (3)º	Block, orange
γ = 73.73 (3)º	0.35 × 0.28 × 0.26 mm
*V* = 643.0 (2) Å^3^	

### Data collection

**Table:**

Bruker APEXII area-detector diffractometer	2937 independent reflections
Radiation source: fine-focus sealed tube	2727 reflections with *I* > 2σ(*I*)
Monochromator: graphite	*R*_int_ = 0.020
*T* = 296(2) K	θ_max_ = 27.5º
ω scans	θ_min_ = 1.9º
Absorption correction: multi-scan(SADABS; Sheldrick, 1996)	*h* = −9→9
*T*_min_ = 0.766, *T*_max_ = 0.814	*k* = −10→11
10449 measured reflections	*l* = −14→13

### Refinement

**Table:**

Refinement on *F*^2^	Secondary atom site location: difference Fourier map
Least-squares matrix: full	Hydrogen site location: inferred from neighbouring sites
*R*[*F*^2^ > 2σ(*F*^2^)] = 0.028	H-atom parameters constrained
*wR*(*F*^2^) = 0.077	*w* = 1/[σ^2^(*F*_o_^2^) + (0.0466*P*)^2^ + 0.1331*P*] where *P* = (*F*_o_^2^ + 2*F*_c_^2^)/3
*S* = 1.00	(Δ/σ)_max_ < 0.001
2937 reflections	Δρ_max_ = 0.32 e Å^−^^3^
160 parameters	Δρ_min_ = −0.17 e Å^−^^3^
Primary atom site location: structure-invariant direct methods	Extinction correction: none

### Special details

**Table:**

Geometry. All e.s.d.'s (except the e.s.d. in the dihedral angle between two l.s. planes) are estimated using the full covariance matrix. The cell e.s.d.'s are taken into account individually in the estimation of e.s.d.'s in distances, angles and torsion angles; correlations between e.s.d.'s in cell parameters are only used when they are defined by crystal symmetry. An approximate (isotropic) treatment of cell e.s.d.'s is used for estimating e.s.d.'s involving l.s. planes.
Refinement. Refinement of *F*^2^ against ALL reflections. The weighted *R*-factor *wR* and goodness of fit *S* are based on *F*^2^, conventional *R*-factors *R* are based on *F*, with *F* set to zero for negative *F*^2^. The threshold expression of *F*^2^ > σ(*F*^2^) is used only for calculating *R*-factors(gt) *etc*. and is not relevant to the choice of reflections for refinement. *R*-factors based on *F*^2^ are statistically about twice as large as those based on *F*, and *R*- factors based on ALL data will be even larger.

### Fractional atomic coordinates and isotropic or equivalent isotropic displacement parameters (Å^2^)

**Table:**

	*x*	*y*	*z*	*U*_iso_*/*U*_eq_	
Ni1	0.5000	0.0000	0.5000	0.03138 (10)	
O1	0.57408 (15)	0.09660 (13)	0.62529 (9)	0.0365 (2)	
O2	0.73982 (18)	0.1105 (2)	0.80263 (12)	0.0627 (4)	
N1	0.22745 (17)	0.14317 (15)	0.55226 (12)	0.0371 (3)	
N2	0.5262 (2)	0.22458 (15)	0.34548 (12)	0.0399 (3)	
C1	0.4656 (2)	0.17196 (17)	0.71970 (13)	0.0346 (3)	
C2	0.5492 (2)	0.1879 (2)	0.81787 (15)	0.0439 (4)	
C3	0.4433 (3)	0.2733 (2)	0.91789 (16)	0.0561 (5)	
H3A	0.5018	0.2827	0.9797	0.067*	
C4	0.2490 (3)	0.3462 (3)	0.92760 (17)	0.0611 (5)	
H4A	0.1790	0.4061	0.9944	0.073*	
C5	0.1622 (3)	0.3292 (2)	0.83891 (16)	0.0502 (4)	
H5A	0.0322	0.3771	0.8463	0.060*	
C6	0.2657 (2)	0.24013 (18)	0.73553 (14)	0.0391 (3)	
C7	0.1632 (2)	0.22884 (19)	0.64567 (15)	0.0404 (3)	
H7A	0.0370	0.2900	0.6562	0.048*	
C8	0.1073 (2)	0.1771 (2)	0.46023 (16)	0.0471 (4)	
H8A	−0.0265	0.2136	0.5026	0.057*	
H8B	0.1226	0.0780	0.4246	0.057*	
C9	0.1672 (3)	0.3098 (2)	0.35454 (18)	0.0560 (5)	
H9A	0.1508	0.4073	0.3926	0.067*	
H9B	0.0809	0.3376	0.2976	0.067*	
C10	0.3716 (3)	0.2666 (2)	0.27522 (15)	0.0496 (4)	
H10A	0.3850	0.1740	0.2322	0.059*	
H10B	0.3914	0.3588	0.2098	0.059*	
C11	0.7109 (3)	0.1849 (2)	0.25224 (16)	0.0520 (4)	
H11A	0.7230	0.2777	0.1876	0.078*	
H11B	0.7164	0.0922	0.2135	0.078*	
H11C	0.8137	0.1590	0.2950	0.078*	
C12	0.5251 (3)	0.3671 (2)	0.40162 (16)	0.0506 (4)	
H12A	0.5361	0.4588	0.3357	0.076*	
H12B	0.6311	0.3405	0.4414	0.076*	
H12C	0.4073	0.3950	0.4644	0.076*	
C13	0.8151 (3)	0.0722 (3)	0.9111 (2)	0.0726 (6)	
H13A	0.9487	0.0188	0.8885	0.109*	
H13B	0.7480	0.0003	0.9744	0.109*	
H13C	0.8001	0.1714	0.9449	0.109*	

### Atomic displacement parameters (Å^2^)

**Table:**

	*U*^11^	*U*^22^	*U*^33^	*U*^12^	*U*^13^	*U*^23^
Ni1	0.03755 (15)	0.03032 (15)	0.02764 (14)	−0.00398 (10)	−0.01031 (10)	−0.00902 (9)
O1	0.0411 (5)	0.0390 (5)	0.0316 (5)	−0.0064 (4)	−0.0097 (4)	−0.0126 (4)
O2	0.0516 (7)	0.1037 (11)	0.0382 (6)	−0.0142 (7)	−0.0142 (5)	−0.0222 (7)
N1	0.0390 (6)	0.0359 (6)	0.0359 (6)	−0.0029 (5)	−0.0125 (5)	−0.0069 (5)
N2	0.0574 (8)	0.0333 (6)	0.0321 (6)	−0.0133 (6)	−0.0123 (5)	−0.0053 (5)
C1	0.0487 (8)	0.0283 (7)	0.0271 (6)	−0.0097 (6)	−0.0087 (6)	−0.0042 (5)
C2	0.0555 (9)	0.0490 (9)	0.0309 (7)	−0.0158 (7)	−0.0100 (6)	−0.0088 (6)
C3	0.0760 (12)	0.0648 (12)	0.0332 (8)	−0.0167 (10)	−0.0136 (8)	−0.0180 (8)
C4	0.0814 (13)	0.0576 (11)	0.0378 (9)	−0.0024 (10)	−0.0058 (8)	−0.0231 (8)
C5	0.0587 (10)	0.0421 (9)	0.0403 (8)	0.0025 (7)	−0.0056 (7)	−0.0134 (7)
C6	0.0506 (8)	0.0301 (7)	0.0328 (7)	−0.0034 (6)	−0.0077 (6)	−0.0072 (6)
C7	0.0409 (7)	0.0342 (7)	0.0404 (8)	0.0014 (6)	−0.0091 (6)	−0.0078 (6)
C8	0.0410 (8)	0.0518 (10)	0.0497 (9)	0.0008 (7)	−0.0196 (7)	−0.0135 (8)
C9	0.0695 (11)	0.0444 (9)	0.0560 (10)	0.0036 (8)	−0.0367 (9)	−0.0040 (8)
C10	0.0780 (12)	0.0395 (8)	0.0349 (8)	−0.0130 (8)	−0.0244 (8)	0.0008 (6)
C11	0.0709 (11)	0.0474 (9)	0.0374 (8)	−0.0225 (8)	−0.0044 (8)	−0.0050 (7)
C12	0.0792 (12)	0.0354 (8)	0.0430 (9)	−0.0203 (8)	−0.0159 (8)	−0.0067 (7)
C13	0.0625 (12)	0.1093 (19)	0.0514 (11)	−0.0211 (12)	−0.0217 (9)	−0.0104 (11)

### Geometric parameters (Å, °)

**Table:**

Ni1—O1	2.0061 (11)	C5—C6	1.416 (2)
Ni1—O1^i^	2.0061 (11)	C5—H5A	0.9300
Ni1—N1	2.0547 (14)	C6—C7	1.439 (2)
Ni1—N1^i^	2.0547 (14)	C7—H7A	0.9300
Ni1—N2^i^	2.3081 (15)	C8—C9	1.518 (3)
Ni1—N2	2.3081 (15)	C8—H8A	0.9700
O1—C1	1.2899 (17)	C8—H8B	0.9700
O2—C2	1.372 (2)	C9—C10	1.520 (3)
O2—C13	1.399 (2)	C9—H9A	0.9700
N1—C7	1.287 (2)	C9—H9B	0.9700
N1—C8	1.467 (2)	C10—H10A	0.9700
N2—C12	1.471 (2)	C10—H10B	0.9700
N2—C11	1.473 (2)	C11—H11A	0.9600
N2—C10	1.487 (2)	C11—H11B	0.9600
C1—C6	1.418 (2)	C11—H11C	0.9600
C1—C2	1.433 (2)	C12—H12A	0.9600
C2—C3	1.375 (2)	C12—H12B	0.9600
C3—C4	1.394 (3)	C12—H12C	0.9600
C3—H3A	0.9300	C13—H13A	0.9600
C4—C5	1.361 (3)	C13—H13B	0.9600
C4—H4A	0.9300	C13—H13C	0.9600
			
O1—Ni1—O1^i^	180.0	C5—C6—C7	117.77 (15)
O1—Ni1—N1	88.00 (5)	C1—C6—C7	122.05 (13)
O1^i^—Ni1—N1	92.00 (5)	N1—C7—C6	126.96 (14)
O1—Ni1—N1^i^	92.00 (5)	N1—C7—H7A	116.5
O1^i^—Ni1—N1^i^	88.00 (5)	C6—C7—H7A	116.5
N1—Ni1—N1^i^	180.00 (7)	N1—C8—C9	108.79 (14)
O1—Ni1—N2^i^	87.10 (5)	N1—C8—H8A	109.9
O1^i^—Ni1—N2^i^	92.90 (5)	C9—C8—H8A	109.9
N1—Ni1—N2^i^	98.96 (6)	N1—C8—H8B	109.9
N1^i^—Ni1—N2^i^	81.04 (6)	C9—C8—H8B	109.9
O1—Ni1—N2	92.90 (5)	H8A—C8—H8B	108.3
O1^i^—Ni1—N2	87.10 (5)	C8—C9—C10	115.86 (14)
N1—Ni1—N2	81.04 (6)	C8—C9—H9A	108.3
N1^i^—Ni1—N2	98.96 (6)	C10—C9—H9A	108.3
N2^i^—Ni1—N2	180.00 (5)	C8—C9—H9B	108.3
C1—O1—Ni1	129.01 (10)	C10—C9—H9B	108.3
C2—O2—C13	117.24 (15)	H9A—C9—H9B	107.4
C7—N1—C8	116.00 (13)	N2—C10—C9	116.40 (13)
C7—N1—Ni1	126.39 (11)	N2—C10—H10A	108.2
C8—N1—Ni1	116.42 (10)	C9—C10—H10A	108.2
C12—N2—C11	107.24 (14)	N2—C10—H10B	108.2
C12—N2—C10	110.49 (14)	C9—C10—H10B	108.2
C11—N2—C10	107.71 (13)	H10A—C10—H10B	107.3
C12—N2—Ni1	110.96 (10)	N2—C11—H11A	109.5
C11—N2—Ni1	108.89 (10)	N2—C11—H11B	109.5
C10—N2—Ni1	111.39 (10)	H11A—C11—H11B	109.5
O1—C1—C6	124.80 (13)	N2—C11—H11C	109.5
O1—C1—C2	118.76 (14)	H11A—C11—H11C	109.5
C6—C1—C2	116.44 (14)	H11B—C11—H11C	109.5
O2—C2—C3	124.23 (16)	N2—C12—H12A	109.5
O2—C2—C1	114.18 (14)	N2—C12—H12B	109.5
C3—C2—C1	121.60 (16)	H12A—C12—H12B	109.5
C2—C3—C4	120.66 (17)	N2—C12—H12C	109.5
C2—C3—H3A	119.7	H12A—C12—H12C	109.5
C4—C3—H3A	119.7	H12B—C12—H12C	109.5
C5—C4—C3	119.69 (16)	O2—C13—H13A	109.5
C5—C4—H4A	120.2	O2—C13—H13B	109.5
C3—C4—H4A	120.2	H13A—C13—H13B	109.5
C4—C5—C6	121.33 (17)	O2—C13—H13C	109.5
C4—C5—H5A	119.3	H13A—C13—H13C	109.5
C6—C5—H5A	119.3	H13B—C13—H13C	109.5
C5—C6—C1	120.12 (15)		

Symmetry codes: (i) −*x*+1, −*y*, −*z*+1.
